# A DOUBLE HYPERAUTOFLUORESCENT RING IN A 33-YEAR-OLD-FEMALE PATIENT

**DOI:** 10.1097/ICB.0000000000001337

**Published:** 2022-09-02

**Authors:** Mariana M. da Palma, Molly Marra, Mark E. Pennesi

**Affiliations:** * Department of Ophthalmology, Casey Eye Institute, Oregon Health and Science University, Portland, Oregon; and; † Department of Ophthalmology and Visual Science, Federal University of São Paulo, São Paulo, Brazil.

**Keywords:** enhanced S-cone syndrome, hereditary eye diseases, *NR2E3*, *NRL*, retinoschisis

## Abstract

To describe the clinical phenotype and molecular diagnosis of a patient with atypical presentation of enhanced S-cone syndrome.

**Methods:**

This is a case report of a patient who underwent best-corrected visual acuity, slit-lamp exam, fundus examination, autofluorescence, optical coherence tomography, kinetic perimetry, and full-field electroretinography. Genetic testing was performed via next-generation sequencing.

**Results:**

A 33-year-old female patient presented with mild nyctalopia, but normal rod function measured by electroretinogram and foveoschisis on optical coherence tomography. She also presented a double hyperautofluorescent ring on autofluorescence. Genetic testing found a pathogenic variant c.925C>G (p.Arg309Gly) and a likely pathogenic variant c.299C>T (p.Arg77Trp) in *NR2E3* gene.

**Conclusion:**

Enhanced S-cone syndrome may present without the pathognomonic findings of decreased rod function on electroretinogram, suggesting the importance of genetic testing in retinal diseases for diagnosis.

## Abstract 2

We report a patient with enhanced S-cone syndrome because of pathogenic (p.Arg309Gly), and likely pathogenic (p.Arg77Trp) variants in the *NR2E3* gene with atypical electroretinogram responses and atypical double hyperautofluorescent ring in both eyes.

## Case Findings

**Fig. 1.**
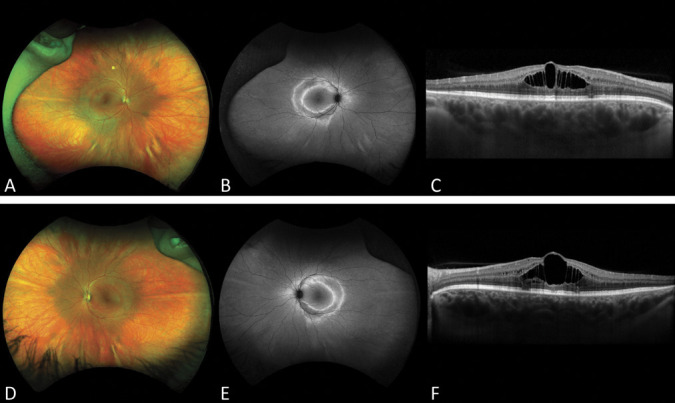
Multimodal retinal imaging. **A** and **D.** Fundus photograph showed blunted foveal reflex (**B** and **E**) autofluorescence showed a double hyperautofluorescent ring. **C** and **F.** Optical coherence tomography showed outer retinal atrophy outside macula area and foveoschisis.

**Fig. 2.**
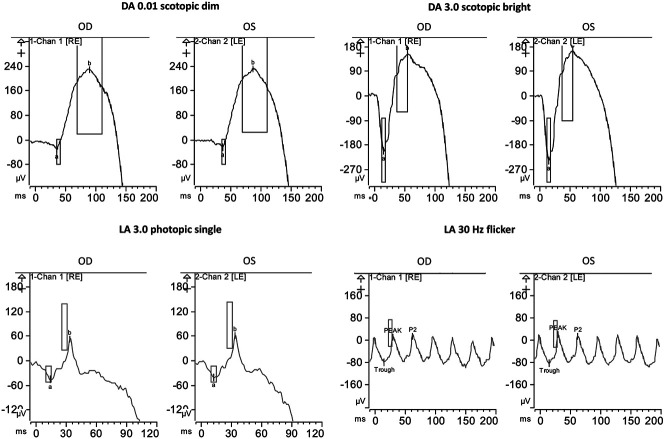
Functional testing. The full-field electroretinogram showed normal amplitudes and normal implicit time of rod-dependent responses and normal amplitude of cone-dependent responses, but prolonged timing of cones.

A 33-year-old female patient was referred to the Ophthalmic Genetics Division at the Casey Eye Institute because of retinal changes and cystoid macular edema according to chart review. There was no family history of inherited retinal diseases. She was complaining of decreased visual acuity, and photopsias. She reported photoaversion since Age 14, and mild nyctalopia since Age 32 causing difficulty driving at night. She was previously diagnosed with Type 1 Von Willebrand's disease. Her best-corrected visual acuity was 20/40 in the right and 20/70 in the left eye. Her refraction was −0.75 + 0.75 × 115 and −0.75 in the right and left eyes, respectively. The fundus examination in both eyes showed clear media, waxy pallor of the optic nerves, tortuous vessels, and a blunted foveal reflex. The autofluorescence showed a double hyperautofluorescent ring and scattered hyperautofluorescent spots beyond the outside ring in both eyes. Optical coherence tomography showed bilateral foveoschisis and outer retinal atrophy outside the macula area (Figure 1). The kinetic visual field showed intact isopters to large targets (V4e, III4e, I4e, and I3e). Mildly constricted isopters to I2e with blind spots to I2e bilaterally and scotoma to I2e in the right eye. The full-field electroretinogram was performed in accordance with the International Society for Clinical Electrophysiology of Vision standards.^1^ The electroretinogram showed normal amplitudes and normal implicit time of rod-dependent responses and normal amplitude of cone-dependent responses, but prolonged timing of cones (Figure 2). These results indicated that the generalized retinal function is mostly normal in both eyes, but there were some mild changes in cone-driven responses reflecting the pericentral loss.

## Differential Diagnosis

Because of pericentral outer retinal thinning on optical coherence tomography associated with double hyperautofluorescent ring closer to the vascular arcades, pericentral retinitis pigmentosa was suggested even with minimal changes on electroretinogram and visual fields. The differential for foveal schisis includes: optic pit maculopathy, myopic retinoschisis, niacin use, and stellate nonhereditary idiopathic foveomacular retinoschisis, and X-linked retinoschisis. She had no history of niacin or taxane use. X-linked retinoschisis in females is uncommon, but has already been described because of lyonization or homozygosity.^2^

## Additional Testing

A panel genetic testing covering 322 genes revealed a heterozygous likely pathogenic variant c.299C > T (p.Arg77Trp), and a heterozygous pathogenic variant c.925C>G (p.Arg309Gly) in *NR2E3.* The second allele was confirmed to be present in her daughter.

## Final Diagnosis

Pathogenic variants in *NR2E3*, located on chromosome 15q22.32, are associated with autosomal recessive enhanced S-cone syndrome (ESCS); Goldman–Favre syndrome; clumped pigmentary retinal degeneration; recessive retinitis pigmentosa; and also, dominant retinitis pigmentosa.^3^ According to HGMD, more than 75 variants have already been described. The majority are missense variants and the majority are associated with ESCS (http://www.hgmd.cf.ac.uk/ac/). The missense variant found in our patient, c.925C>G (p.Arg309Gly) has been previously described^4^ in a heterozygous state in patients with ESCS^4,5^ and homozygous in a 9-year-old girl with ESCS.^6^ The c.299C>T (p.Arg77Trp) variant, has been reported in a compound heterozygous state in a patient with ESCS.^7^ *NR2E3* is composed of eight exons. The variation p.Arg77Trp is located on exon 3, and p.Arg309Gly is located on exon 6, the same exon where the frequent variant Arg311Gln is located.^3^ *NR2E3* plays an important role in retinal photoreceptor differentiation and it is expressed in the outer nuclear layer. Pathogenic variants in *NR2E3* result in defects of photoreceptor differentiation, whereby cells destined to a rod fate develop into S-cones.^8^ It has been shown that the variant p.Arg309Gly reduces protein stability,^9^ and interaction with *NRL* and *CRX genes*.^4^

## Treatment and Follow-Up

Topical and oral carbonic anhydrase inhibitors were reported to be successful in treating schisis in ESCS.^10^ The patient was on acetazolamide extended-release oral capsules (Diamox Sequels) 500 mg per-day.

## Discussion

Patients with ESCS typically complain of nyctalopia from an early age and decreased central vision. Hyperopic refractive errors have been previously reported. Patients may also have progressive foveal schisis. Peripheral retinoschisis can also be found. Nummular pigment clumping along vascular arcades is common at fundus examination. Fundus autofluorescence shows hypoautofluorescence outside the arcades because of photoreceptor loss.^3–8^ A hyperautofluorescent ring has already been described between the area of hypoautofluorescence and the macula.^8^ The electroretinogram findings are pathognomonic for this condition,^8,11,12^ typically demonstrating complete loss of the rod-driven responses, delayed responses to single flashe photopic stimuli with simplified waveforms, and markedly delayed and attenuated responses to 30-Hz flicker. These findings suggest responses driven only by the supernormal S-cone population.^11,12^ In patients with ESCS, recordings with an orange background demonstrate a supranormal response to short-wavelength (blue) stimuli reflecting supernormal S-cone function,^8^ but we were unable to obtain this testing.

In a normal retina, there are approximately 6 million cones and 125 million rods. Cones are divided into three subtypes: short-wavelength cone sensitive (S-cone or blue cone), middle-wavelength sensitive (M-cone or green cone), and long-wavelength sensitive (L-cone or red cone). In enhanced S-cone syndrome, there is an excess number of S-cones in the retina,^3^ whereas in an adult retina, S-cones represent only 8%–10% of cone population.^13^ Histopathologic analysis of a postmortem retina from a patient with enhanced S-cone syndrome showed that 92% of the cones were S-cones.^14^ To the best of our knowledge, this is the first description of enhanced S-cone syndrome with normal rod function and only mild delayed cone responses. The patient presented with the typical macular schisis without rod dysfunction or pigmentary retinal changes and a double hyperautofluorescent ring. These autofluorescence changes have already been described in patients with autosomal dominant NR2E3-related retinitis pigmentosa because of a pathogenic variant p.Gly56Arg located on exon 2.^15^ The outer ring extends toward the periphery in a centripetal fashion ultimately becoming hypoautofluorescent. The inner perimacular ring extends centrifugally. The area between the hyperautofluorescent rings denotes the area where photoreceptors have degenerated.^15^ Panel-based genetic testing focusing on genes associated with inherited retinal diseases allowed an accurate diagnosis in this case. The presentation of the disease differs from the classic descriptions of ESCS, and demonstrates the need for genetic testing to obtain an accurate diagnosis. The presence of macular schisis and a double ring on autofluoresence, biallelic *NR2E3* mutations should be considered.
